# Emotivity in the Voice: Prosodic, Lexical, and Cultural Appraisal of Complaining Speech

**DOI:** 10.3389/fpsyg.2020.619222

**Published:** 2021-01-18

**Authors:** Maël Mauchand, Marc D. Pell

**Affiliations:** School of Communication Sciences and Disorders, McGill University, Montreal, QC, Canada

**Keywords:** pragmatics, cross-cultural, vocal affect, emotive involvement, complaint

## Abstract

Emotive speech is a social act in which a speaker displays emotional signals with a specific intention; in the case of third-party complaints, this intention is to elicit empathy in the listener. The present study assessed how the emotivity of complaints was perceived in various conditions. Participants listened to short statements describing painful or neutral situations, spoken with a complaining or neutral prosody, and evaluated how complaining the speaker sounded. In addition to manipulating features of the message, social-affiliative factors which could influence complaint perception were varied by adopting a cross-cultural design: participants were either Québécois (French Canadian) or French and listened to utterances expressed by both cultural groups. The presence of a complaining tone of voice had the largest effect on participant evaluations, while the nature of statements had a significant, but smaller influence. Marginal effects of culture on explicit evaluation of complaints were found. A multiple mediation analysis suggested that mean fundamental frequency was the main prosodic signal that participants relied on to detect complaints, though most of the prosody effect could not be linearly explained by acoustic parameters. These results highlight a tacit agreement between speaker and listener: what characterizes a complaint is how it is said (i.e., the tone of voice), more than what it is about or who produces it. More generally, the study emphasizes the central importance of prosody in expressive speech acts such as complaints, which are designed to strengthen social bonds and supportive responses in interactive behavior. This intentional and interpersonal aspect in the communication of emotions needs to be further considered in research on affect and communication.

## Introduction

While the role of the voice in social interaction has been receiving growing interest over the last decades, literature on the topic has been scattered across research fields. On the one hand, experimental psychology has been focusing on affective speech and emotions (Frick, 1985; Scherer, 2003; Juslin, 2013); on the other hand, intentionality, speech acts, and attitudes have been mostly addressed by pragmatics and theoretical linguistics (Searle, 1965; Grice, 1989; Wichmann, 2002; Culpeper and Terkourafi, 2017). A large part of our daily social interactions is inherently *emotive*, relying on the attitudinal, intentional use of emotional signals (Caffi and Janney, 1994). These interactions, involving both speaker and listener in a complex collaborative timeline, remain poorly understood. The nature and components of emotive interactions can be investigated through an intersectional approach, embedding social and affective psychology methods into the theoretical pragmatics framework of emotivity through the Emotions As Social Information (EASI) model (Van Kleef, 2009). Focusing on the case of complaints, the present study examines how emotivity is conveyed through speech, and how affective signals in the voice are processed in different social and cultural contexts.

Complaints are intentional verbal expressions of social pain, distress, or displeasure (Boxer, 1993; Drew, 1998; Laforest, 2002), and are usually divided in two categories. *Direct* complaints are addressed directly to the source of the issue, with the purpose of terminating or solving the issue (Trenchs, 1994; Laforest, 2002). The present study focuses on *indirect* or *third-party* complaints, which are addressed to a third party usually unrelated to the issue (Drew, 1998; Edwards, 2005). Third-party complaints are non-instrumental in nature (Alicke et al., 1992); they do not aim to solve the problem they address, but have a more indirect function of promoting social affiliation though affectivity and empathy (Drew and Walker, 2009; Ogden, 2010). In what follows, the term complaint will be used to refer exclusively to third-party complaints.

The social importance of complaints is implied by their frequency; it is said that individuals complain more than four times a day on average (Alicke et al., 1992). While many types of speech acts can lead to a strengthening of social bonds, complaints appear to directly serve this purpose; this is accomplished through long, interactive sequences in which the complainer negotiates the affiliation of their listener (Drew and Walker, 2009; Selting, 2010). Complaints are usually defined by tightly bounded topics with a clear beginning and end (Drew, 1998), often used as ice-breakers or conversation openers (Boxer, 1993; Kowalski, 2002). Complainers may open with an initial complaint to probe the affiliative response of their listener, which will determine the course of the negotiation (Traverso, 2009). Ultimately, it is the listener who chooses whether or not to collaborate and affiliate with the speaker (Edwards, 2005; Selting, 2010).

Beyond describing a negative situation, a core function of complaints is to provide evidence of how the speaker *feels* about the situation (Drew, 1998). In order to gain affiliation, a complaint should allow the listener to share the affective state of the speaker and empathize with them (Acuña-Ferreira, 2002; Edwards, 2005). Since most complaints describe a past event involving felt pain or distress, it is unlikely that the speaker is fully experiencing these emotions as they complain; rather, these expressions may be viewed as instances of “reconstructed affect” (Selting, 2010). Complaining is thus an *emotive* or *expressive* speech act (Scarantino, 2017), a type of social performance in which the speaker intentionally displays affective markers to achieve interactive goals (Caffi and Janney, 1994; Acuña-Ferreira, 2002). These markers are the negotiating products of a complaint, informing the listener of the complainer’s emotions (Edwards, 2005) and sharing these emotions through mood contagion (Kowalski, 2002). The affective component of a complaint is usually more important than the object of the complaint itself, from which the interaction can drift off while remaining a complaining collaboration (Edwards, 2005; Traverso, 2009).

The Emotions as Social Information (EASI) model (Van Kleef, 2009) provides a useful framework for investigating the perceptual and social dimensions of complaining speech in greater depth. The EASI model emphasizes that affective displays are more than biological symptoms and can be used to influence others by triggering inferential processes and affective reactions. For complaints to succeed (i.e., promote social affiliation and strengthen bonds), complainers and listeners need to effectively display, perceive and respond to communicative signals of affect and emotivity, which are frequently marked through a complainer’s voice, or *speech prosody*. Here, we refer to prosody as suprasegmental acoustic features of speech - pitch, loudness, voice quality, rhythm - that speakers modulate, intentionally or not, to express meanings, emotions, and attitudes in their voice (Pell, 2001; Scherer, 2003). The manner in which prosody is used in complaining interactions and its impact on listeners has seldom been explored.

According to Brunswikian lens models of speech, the emotions of speakers are encoded by a constellation of acoustic cues that are then decoded by listeners into emotional representations (Brunswik, 1956; Grandjean et al., 2006; Laukka et al., 2016). A number of studies have reported that vocal expressions of basic emotion (e.g., anger, sadness, happiness) show specific patterns of pitch, loudness, rhythm, and voice quality that yield successful recognition of these emotions by listeners (see Juslin and Laukka, 2003 and Scherer and Bänziger, 2004 for reviews). However, vocal changes are not always symptoms of the speaker’s internal emotional state; for example, prosody can be intentionally used as an expressive device to elicit empathy in the listener, allowing interactants to experience (Aziz-Zadeh et al., 2010; Rodero, 2011) and understand (Regenbogen et al., 2012; Ong et al., 2018) the speaker’s feelings. This combination of affective and inferential processing of prosody provides the speaker with important emotional influence and bolsters supportive behavior from the listener, with potential social benefits for both parties (Van Kleef, 2009; Pell and Kotz, 2020).

It has been reported that prosodic features of complaints signal increased affectivity through elevated mean fundamental frequency and frequency variability, syllable elongation, and emphatic accentuations (Acuña-Ferreira, 2002; Ogden, 2010; Selting, 2010; Rao, 2013). In emotional contexts, these acoustic changes are often associated with negative and high arousal emotions, like anger, sadness, surprise and indignation (Drew, 1998; Selting, 2010). Complaints may also be viewed as expressions of pain and suffering, which are associated with specific forms of vocal expression (Lerner et al., 2016; Raine et al., 2019). The present study is based on a large set of complaining utterances that display many of the acoustic and emotional properties described above, as well as voice quality patterns that resemble expressions of simulated pain (Mauchand and Pell, n.d. a, under review; Raine et al., 2019).

While the role of prosody in communicating the emotive involvement of complainers is heavily suggested, most of the literature on complaints comes from the pragmatics field, based largely on descriptive and qualitative analyses of conversations (Boxer, 1993; Drew, 1998; Acuña-Ferreira, 2002; Edwards, 2005; Traverso, 2009; Ogden, 2010; Selting, 2010; Rao, 2013). No experimental investigation has been conducted to establish how prosody affects the perception of complaints, especially with respect to other lexical or contextual cues that complainers often provide. As mentioned above, the emotive involvement of the speaker is often more important than the object of the complaint, meaning that even innocuous topics can be the focus of valid complaints (Boxer, 1993). Still, the preference of complainers to provide specific descriptions (Alicke et al., 1992), expletives (Drew, 1998), and extreme-case formulation (Selting, 2010) suggest that complaining emotive interactions depend on both linguistic and paralinguistic cues, albeit in an unclear manner.

The integration of prosodic and verbal affective signals and their combined impacts on social perception can be complex. The relative effects of cues in each channel may vary at different stages of perception, processing, and evaluation (Paulmann and Kotz, 2008; Pell et al., 2011; Meconi et al., 2018), and likely depend heavily on task demands (Regenbogen et al., 2012) and the emotional salience of cues (Wambacq and Jerger, 2004). In expressive speech acts, the role of prosody is traditionally described as an indirect, illocutionary force that can only convey meaning with the appropriate verbal statement (Grice, 1989; Wichmann, 2000). However, recent studies suggest that prosody alone can reveal the intentions of a speaker in a powerful manner (Hellbernd and Sammler, 2016; Caballero et al., 2018; Truesdale and Pell, 2018). For example, in motivating and persuasive speech, prosody can “tag” verbal information as important and increase the persuasiveness of a speaker even when the verbal information is not credible (Zougkou et al., 2017; Van Zant and Berger, 2020). Prosody is thus an important emotive *and* persuasive device in low-involvement communicative situations (Gelinas-Chebat and Chebat, 1992), which is often the case of third-party complaints (Alicke et al., 1992; Boxer, 1993).

The use of affect as social information further depends on a number of social-relational factors, such as cultural display rules, familiarity, or group biases (Van Kleef, 2009). Indeed, if the traditional view of emotions as genuine biological responses could imply a universal consistency in their expression (Frick, 1985; Ekman et al., 1987), describing affective displays as social tools implies investigating how social and cultural contexts affect their usage (Van Kleef, 2009; Scarantino, 2017). Several studies already suggest that despite a basic universality, emotional communication can be affected by cultural in-group advantages (Elfenbein and Ambady, 2002), depend on cultural proximity (Laukka et al., 2016) and seem to mainly affect positive rather than negative emotions (Sauter et al., 2010; Scherer et al., 2011; see Laukka and Elfenbein, 2020 for a review). Often, out-group accent perception does not impede how well emotions are recognized but does affect perceived intensity, empathic arousal or physiological responses from listeners (Soto and Levenson, 2009; Mac et al., 2010; Thierry et al., 2015). Beyond emotions, a speaker’s accent is a marker of identity: the information (or lack thereof) that it carries is known to interfere with speech processing (Floccia et al., 2006; Sumner and Samuel, 2009), create biases and stereotypes (Kuiper, 2005; Lev-Ari and Keysar, 2010; Heblich et al., 2015), and affect the appraisal of diverse pragmatic cues (Yuan et al., 2019; Jiang et al., 2020). Cultural factors may thus affect numerous stages of production, perception, and interpretation of emotive speech.

Complaining appears to be a convention rooted in a number of cultures. Be it the French *se plaindre* (Traverso, 2009), the Australian *whinge* (Edwards, 2005), the German *Jammern* (Winchatz, 2016), or the Israeli *kiturim* (Katriel, 2013), many societies have defined complaining as a cultural custom, each with their own specificities and social implications. These potential cultural specificities raise the question of what constitutes a complaint across cultures. Yet, few studies have directly investigated the cross-cultural aspect of complaints. An investigation by Rao (2013) reported that Mexican Spanish complaints showed intonational variation typical of European Spanish complaints, but in a more accentuated manner. Similarly, Mauchand and Pell, n.d. b, under review reported that Canadian French (Québécois) and European French complaints show strong acoustic similarities but sometimes differ in the weight given to certain prosodic cues and the emotional representations they convey. Parallel work on direct complaints also show some pragmatic differences between native and non-native complaints (Trenchs, 1994; Kraft and Geluykens, 2002). Beyond the definition of complaints, these acoustic differences could affect cross-cultural understanding of complaining speech, individuals being potentially more sensitive to emotive prosodic signals from their own group. To date, work which sheds light on these questions has not been undertaken.

The goal of the present study was to give insight on how third-party complaints are perceived from affective prosody and other cues that mark a speaker’s “complaining intentions,” using the Emotions as Social Information (EASI) model as a general reference (Van Kleef, 2009). Furthermore, we explored the role of social-relational variables in this context by studying two francophone groups: French (i.e., European French) and Québécois (i.e., French Canadian). While mutually intelligible, these two groups have different cultural backgrounds and distinct accents, thus allowing the isolation of cultural group (dis)advantages in the processing of complaints in the absence of language barriers. French and Québécois participants listened to pre-validated utterances that varied in prosody, verbal content, and speaker accent, and evaluated “how complaining” each utterance sounded. The study also investigated the relationship between encoding and decoding processes by analyzing how the perception of expressive speech acts, such as complaints, is driven by particular acoustic features of vocal affect signals. It was predicted that a speaker’s tone of voice would be the main marker of a complaining intention, especially when verbal cues did not convey high emotive involvement, i.e., when speakers complained about innocuous rather than explicitly pain-related topics. The detection of complaints was expected to depend on how the speakers produced emotive signals, especially through modulation of voice pitch and other emotion-related cues, which are likely to mediate the effect of complaining prosody on participant’s evaluations. Finally, it was predicted that social-relational factors would influence complaint perception: participants were expected to discriminate complaints from neutral utterances better for speakers of their own cultural group, potentially because of underlying biases and/or specific display rules associated with complaining speech.

## Materials and Methods

### Participants

Power analyses for mixed models (Judd et al., 2016) were performed to determine the required number of participants. Large effects of prosody and verbal content were reported in previous studies with similar procedures (Caballero et al., 2018; Mauchand et al., 2020). Due to the large number of stimuli (*n* = 320), less than 25 participants were required to attain power over 99% for these effects. The effect of culture, if present, would be smaller based on previous cross-cultural studies that have used recognition tasks (Elfenbein and Ambady, 2002; Liu et al., 2015; Laukka et al., 2016; Jiang et al., 2018). Based on an effect size of 0.3 with intercepts and slopes variances of 0.1, a minimum sample size of 57 participants would be required to achieve power of 90% for this variable.

In total, 31 French and 27 Québécois participants, aged 18–35, with no hearing or neurological impairment were recruited in the Montréal area. French participants were born in France, had lived in France until at least 18, and had arrived less than 3 years ago in Montréal (for study or work). Québécois participants were born and lived in Québec (a French-speaking province in Canada) until age 18 and had never lived in France or another francophone country. All participants spoke French as their mother tongue.

Data about participants’ personality and cultural attitudes were collected through a number of tests and questionnaires (see Mauchand and Pell, n.d. a, under review for a full report on these measures). Accent-based implicit biases were measured through a modified Implicit Association Test (Greenwald et al., 1998) consisting of Pleasant and Unpleasant words presented together with French and Québécois neutral utterances (Mauchand and Pell, n.d. a, under review). Explicit attitudes toward French and Québécois populations were probed through a questionnaire based on the Stereotype Content Model (Fiske et al., 2002), composed of 20 questions about the perceived Warmth and Competence of each community. Finally, empathic abilities were assessed through the Perspective-Taking and Empathic Concern subscales of the French version of the Interpersonal Reactivity Index (Gilet et al., 2013).

### Materials

Materials were created and validated in a previous study focusing on the acoustic dimensions of speech complaints (Mauchand and Pell, n.d. b, under review). Stimuli were short spoken utterances describing a past event, constructed in the form of *token sets* (each composed of 4 unique utterances). A token set was built around a root sentence that was manipulated in two ways. First, we modified the verbal content by modifying the last word of the statement, to refer to a neutral event, e.g., “Il a dit que j’étais sorti/He said I was outside” (*Control* condition) or a socially painful event for the speaker, e.g., “Il a dit que j’étais stupide/He said I was stupid” (*Pain* condition). The list of sentences, together with their English translation, can be found in the Appendix. For each type of statement, we then manipulated the form of prosodic expression: speakers uttered each sentence in a manner as if simply reporting the event (*Neutral* condition) or as if complaining to a friend (*Complaint* condition). One token set was thus composed of 4 utterances with different Statement/Prosody combinations: *Control/Neutral, Control/Complaint, Pain/Neutral, Pain/Complaint.* Forty-two token sets were thereby created.

Initially, 672 utterances were produced by 4 French and 4 Québécois speakers (2 males and 2 females in each group) in order to modulate accent/sociocultural features of the stimuli. Recordings were digitally captured in a sound-attenuated chamber with a high-quality head-mounted microphone onto a Tascam recorder (sampling rate of 44.1 kHz, 16-bit, mono, wav format). They were then edited in Praat (Boersma and van Heuven, 2001) into short.wav audio files and normalized to a peak intensity of 70 dB.

A short validation study was conducted to ensure the quality of the recordings and to select a subset of the stimuli for the current study. Ten French (5 males, 5 females, age: *M* = 21.1, sd = 3.8) and 9 Québécois (3 males, 6 females, age: *M* = 23.00, sd = 2.78) participants listened to all utterances from their own group (*n* = 336) and evaluated: (1) whether an utterance was a complaint (yes/no); and (2) if it was a complaint, its intensity of expression on a 5-point scale. Results of the validation task are displayed in Table 1. Pain/Complaint utterances were almost unanimously considered complaints with high intensity ratings, while Control/Neutral utterances were very rarely considered complaints. Results for Pain/Neutral and Control/Pain utterances suggest that prosody had a larger impact than statement type on the perception of complaints.

**TABLE 1:** Results of the validation/selection task, by speaker group (mean + standard deviation).

Utterance/	Unselected utterances		Selected utterances	
prosody type	(*n* = 352)		(*n* = 320)	
	Proportion of YES answers to “is the person complaining?”	Intensity rating^*a*^ (1 to 5)	Proportion of YES answers to “is the person complaining?”	Intensity rating^*a*^ (1 to 5)
***Québécois***				
Control/Neutral	0.26 (0.27)	1.80 (0.87)	0.21 (0.20)	1.89 (0.99)
Control/Complaint	0.81 (0.21)	2.94 (0.91)	0.81 (0.19)	2.89 (0.70)
Pain/Neutral	0.60 (0.21)	1.74 (0.63)	0.64 (0.21)	1.69 (0.63)
Pain/Complaint	0.96 (0.07)	3.15 (0.84)	0.98 (0.05)	3.34 (0.57)
***French***				
Control/Neutral	0.23 (0.16)	1.53 (0.54)	0.25 (0.15)	1.43 (0.45)
Control/Complaint	0.86 (0.13)	3.45 (0.78)	0.87 (0.13)	3.37 (0.62)
Pain/Neutral	0.57 (0.16)	1.64 (0.38)	0.53 (0.18)	1.67 (0.43)
Pain/Complaint	0.95 (0.06)	3.47 (0.60)	0.94 (0.08)	3.46 (0.52)

*^*a*^Note that the rating is only made when answering YES to the previous question.*

For the present study, a subset of utterances was selected to minimize the repetition of sentences in the experiment, to remove potential outliers, and to ensure that stimuli were representative of the speakers’ intentions (complaining vs. neutral) according to listeners from their own group. For each speaker, a token set was selected if there was enough consensus that the Control/Neutral utterance was NOT a complaint and that the Pain/Complaint utterance was indeed a complaint with high intensity ratings. To avoid selection bias on the prosody/statement effects, results for “incongruent” utterances were not taken into account for the selection. Moreover, each speaker had a “mirror” speaker (of the same sex) in the other cultural group that uttered exactly the same token sets, such that each token set was present exactly once in each group. This selection process did not affect the overall perceptual quality of the stimulus set, as scores for selected and unselected items remained close. In total, there were 2 Accents × 4 Speakers × 10 Token Sets × 2 Prosodies × 2 Statements = 320 selected utterances.

Acoustic measures for each of the 320 selected utterances were collected using the Geneva Minimalistic Acoustic Parameter Set/GeMAPS (Eyben et al., 2016) package from the publicly available openSMILE toolkit (Eyben et al., 2010). The GeMAPS constitutes a reliable standardized baseline set of affect-related acoustic measures (for more details on the computation and implementation of the measures, see Eyben et al., 2016). A full acoustic analysis of all 672 stimuli is presented in a previous study (Mauchand and Pell, n.d. a, under review). The present study focuses on measures of pitch, voice quality, and rhythm known to be perceptually relevant in complaint production (Acuña-Ferreira, 2002; Rao, 2013) and other related modes of emotional expression (Laukka et al., 2016; Raine et al., 2019). Note that since the volume of stimuli was normalized for perception, intensity-related acoustic measures could not be reliably extracted for consideration in the present study. The following acoustic measures were computed as a mean measure over the full duration of each utterance:

• F0, the fundamental frequency, indexing pitch on a logarithmic semitone scale. Considering the importance of pitch in complaints, both the mean (F0 M) and the rescaled standard deviation (F0SD) over the utterance were computed.
• Jitter, indexing aperiodicity (instability) of the F0 signal – voice “creakiness”
• Shimmer, the difference of the peak amplitudes of consecutive F0 periods, indexing voice roughness in dB
• Harmonics-to-Noise Ratio (HNR), indexing the relative amount of additive noise in the voice
• F1, first formant center frequency in Hertz
• Utterance duration and final word duration in seconds (computed on Praat).

These measures are summarized in Table 2.

**TABLE 2:** Summary of acoustic measures for the selected stimuli for each speaker group (Mean + standard deviation).

	French		Québécois	
	Neutral	Complaint	Neutral	Complaint
F0 M	28.43 (4.43)	34.13 (3.57)	28.93 (5.23)	34.28 (6.14)
F0 SD	0.14 (0.06)	0.15 (0.08)	0.17 (0.09)	0.20 (0.08)
HNR	6.64 (2.4)	9.22 (1.66)	6.40 (2.59)	7.83 (2.94)
Jitter	0.05 (0.02)	0.04 (0.02)	0.06 (0.03)	0.05 (0.03)
Shimmer	1.31 (0.42)	1.06 (0.35)	1.35 (0.44)	1.18 (0.34)
F1	535.98 (87.65)	554.86 (74.77)	534.44 (61.28)	559.84 (60.59)
Duration	1.16 (0.26)	1.30 (0.30)	1.42 (0.29)	1.46 (0.39)
Final word duration	0.38 (0.12)	0.45 (0.14)	0.47 (0.15)	0.56 (0.17)

*F0 M, mean fundamental frequency; F0 SD, rescaled standard deviation of the fundamental frequency; HNR, Harmonics-to-Noise Ratio; F1, first formant center frequency.*

### Procedure

Each participant was presented all 320 selected stimuli in a fully randomized order using Cedrus Superlab 5 software. The stimuli were divided in 8 blocks of 40 utterances, with a self-monitored break between each block. After presentation of an utterance, participants answered the question “À quel point cette personne est-elle en train de se plaindre?” (*How much is this person complaining?*) on a 7-point Likert scale ranging from *Pas du tout* (*Not at all*) to *Énormément* (*Very much*) by pressing a button on a response box. No time limit was set. Participants were not given any indication or strategy on how to form their answer and were told that there was no right or wrong answer. The whole experiment lasted a little more than 1 h.

## Results

### Main Model

Participant’s ratings were analyzed through a Linear Mixed Effect Model using the R package lme4 (Bates et al., 2015). *T*-tests and *p*-values were computed with Satterthwaite’s approximation using lmerTest package (Kuznetsova et al., 2017). The model was built with the participant’s Response (0–6) as the response variable, and Participant Culture (French/Québécois), Speaker Accent (French/Québécois), Statement (Control/Pain), and Prosody (Neutral/Complaint) as predictors. All 2- and 3-way interactions were also entered as predictor terms. Participant and Speaker/TokenSet were added as random intercepts: TokenSet was nested within Speaker, such that Speaker was one random intercept and the interaction between Speaker and TokenSet was another random intercept, thus accounting for the variability of speakers and the variability of token sets within each speaker. Additionally, Culture, Statement and Prosody were added as uncorrelated by-Speaker/TokenSet slopes, and Accent, Statement and Prosody were added as uncorrelated by-Participant slopes.

**FIGURE 1:**
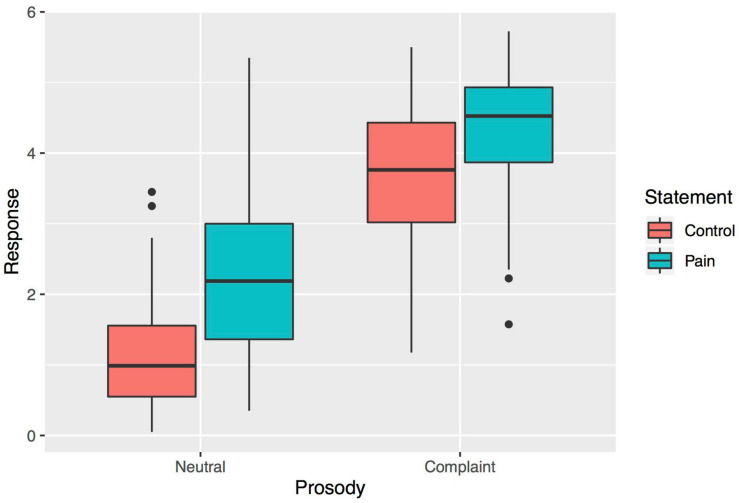
Box-plot summary of Prosody and Statement effects, averaged by participant.

The model accounted for 70% of the variance in the data (*r*^2^ = 0.70). The model revealed a significant effect of Content (β = 0.91, se = 0.13, *t*(19.13) = 7.04, *p* < 0.001), suggesting that when speakers provided linguistic evidence of a painful situation (Pain vs. Control statement), ratings increased by almost 1 point on the scale. A larger effect of Prosody was observed (β = 2.38, se = 0.21, *t*(12.82) = 11.38, *p* < 0.001); statements expressed in a complaining versus neutral tone tended to increase ratings by more than 2 points. Speaker accent was associated with a marginal, yet noticeable effect, as statements produced in the Québécois accent tended to be rated stronger exemplars of complaints than those produced in the French accent (β = 0.68, se = 0.30, *t*(6.20) = 2.27, *p* = 0.063). This trend was informed by another marginally significant pattern in the data, representing a 3-way interaction of Participant Culture, Speaker Accent and Prosody (β = 0.35, se = 0.18, *t*(13.71) = 1.98, *p* = 0.068). The effects of Prosody (Complaint > Neutral) on complaint perception tended to be greater when Québécois participants were listening to the Québécois accent. No other term showed a significant effect (ps > 0.1). Results are summarized in Table 3; Content and Prosody effects are detailed in Figure 1.

**TABLE 3:** Mean rating of “how much the speaker is complaining” by French and Québécois listeners, according to the speaker’s accent, prosody, and the type of statement (0–6 scale).

			French participants	Québécois participants
Accent	Statement	Prosody	M (SD)	M (SD)
French	Control	Neutral	0.92 (0.66)	0.89 (0.63)
		Complaint	3.28 (0.85)	3.27 (1.03)
	Pain	Neutral	1.94 (0.95)	1.70 (1.10)
		Complaint	3.96 (0.74)	3.91 (0.79)
Québécois	Control	Neutral	1.32 (0.72)	1.22 (0.77)
		Complaint	3.88 (0.85)	4.22 (1.07)
	Pain	Neutral	2.72 (0.85)	2.37 (1.01)
		Complaint	4.76 (0.62)	4.90 (0.62)

**FIGURE 2:**
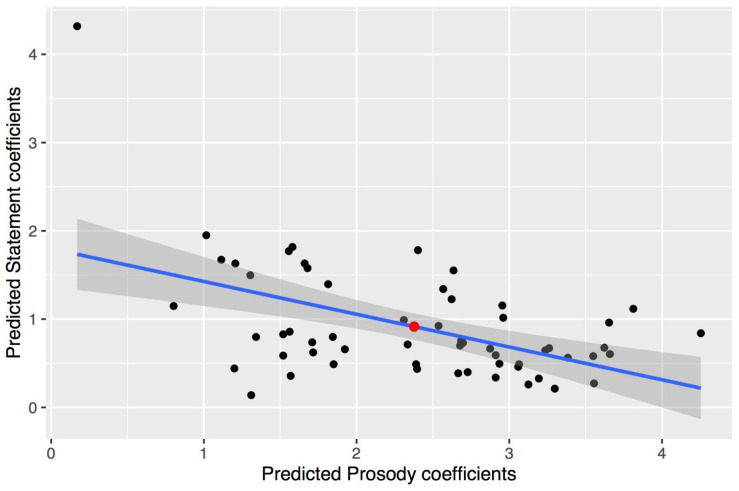
Relationship and linear regression slope between each participant’s predicted coefficients for Statement and Prosody effects in the linear mixed-effects model. The red point indexes the actual coefficients from the whole model.

Follow-up analyses were run to further investigate the relative effects of lexical and prosodic manipulations on the participant’s responses. Looking at the model’s random slopes reveals important variance in these two predictors (0.82 for the slope of Prosody by Participant, 0.45 for the slope of Statement by Participant). A large negative correlation between the two slopes was found (*r* = −0.52), indicating that participants with greater Prosody coefficients tended to have smaller Statement coefficients (see Figure 2). Note that a possible outlier showing extreme coefficients can be seen on Figure 2 but removing this participant from the analysis did not affect results. Correlations were then calculated between the predicted random effects of Prosody and Content and IRI scores, revealing a medium correlation between a participant’s predicted Prosody effect and their score on the Perspective Taking scale (*r* = 0.21), but not the Empathic Concern scale (*r* = 0.06). This pattern was mirrored in correlations with the predicted Statement effect, although to a much lesser extent (PT scale: *r* = −0.13; EC scale: *r* = −0.01). These results suggest that participants who were more sensitive to complaining prosody (especially those with greater perspective-taking skills) relied less on the actual statements.

Analyses also probed the effect of speaker accent and assessed whether this effect could be driven by more specific social-relational factors such as cultural attitudes. A measure of implicit cultural bias was derived from a customized version of the IAT, designed to measure implicit attitudes held by French and Québécois toward speakers of each group (Greenwald et al., 1998; Mauchand and Pell, n.d. a, under review) Based on the IAT D-score, the predicted random effects for each participant showed no particular relationship with implicit biases toward speakers of each cultural group (*r* = 0.04) nor with any of the Stereotype Content Model scores for either group (French Warmth: *r* = −0.05; French Competence: *r* = 0.04; Québécois Warmth: *r* = 0.10; Québécois Competence: *r* = 0.07). This suggests that accent effects were not strongly driven by implicit or explicit cultural biases.

### Mediation of Prosody Effects by Acoustic Parameters

To determine how the effect of prosody on complaint perception relates to specific acoustic properties of the voice, a regression-based mediation analysis with multiple mediators was run following Vanderweele and Vansteelandt (2014). This method accounts for potential relationships between mediators and prevents any effect overlap and redundancies of running several individual mediation analyses. Acoustic parameters described in the Methods section were selected as mediators (see Table 2). The measures from each utterance were standardized by subtracting the mean and dividing by the standard deviation of all utterances. The mediation analysis was thus performed with Prosody as the treatment variable, Response as the outcome variable, and the eight acoustic parameters as mediators.

First, to assess how the treatment variable Prosody affected the mediators, eight linear regressions were run, each with a mediator as the response variable and Prosody as the predictor. Then, to evaluate the effects of the treatment and mediators on the outcome, a multiple linear regression was run with Response as the response variable and the treatment (Prosody) and all eight mediators as predictors. The direct effect of Prosody is given by its coefficient in the latter regression model; the indirect effect of Prosody through a given mediator is given by the product of the effect of Prosody on this mediator and the effect of the mediator on the Response; the total indirect effect of prosody is given by the sum of all such mediated effects.

**FIGURE 3:**
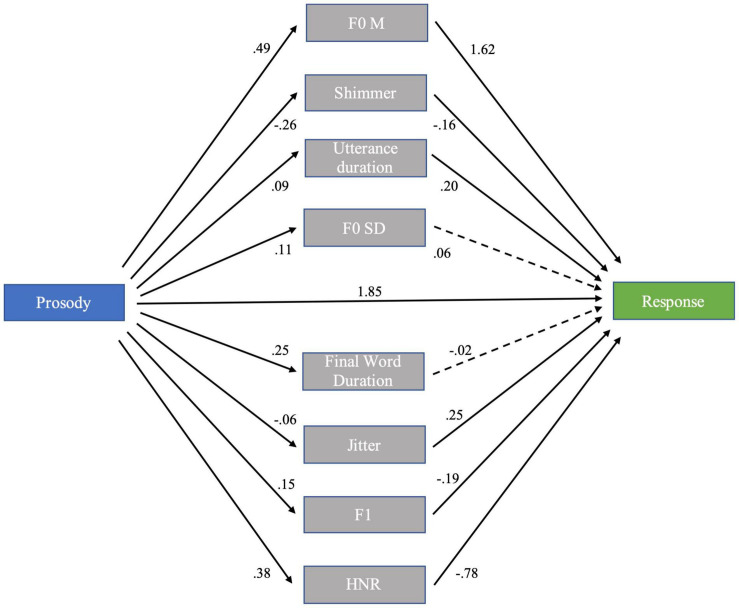
Model summary for the multiple mediation analysis. F0M, mean fundamental frequency; HNR, Harmonics-to-Noise Ratio; F0SD, standard deviation of the fundamental frequency.

The speaker’s mode of prosodic expression had significant effects on each mediator: compared to neutral statements, complaints showed increased F0M (β = 0.49, se < 0.01, *t* = 76.79, *p* < 0.001), increased F0SD (β = 0.11, se < 0.01, *t* = 15.39, *p* < 0.001), reduced shimmer (β = −0.26, se < 0.01, *t* = −37.16, *p* < 0.001), reduced jitter (β = −0.06, se < 0.01, *t* = −8.39, *p* < 0.001), longer utterance duration ((β = 0.09, se < 0.01, *t* = 17.91, *p* < 0.001), and final word duration (β = 0.25, se < 0.01, *t* = 34.56, *p* < 0.001), increased HNR (β = 0.38, se < 0.01, *t* = 55.22, *p* < 0.001), and increased F1 (β = 0.15, se < 0.01, *t* = 21.09, *p* < 0.001). In turn, participant’s Response/ratings were positively affected by F0M (β = 1.62, se = 0.05, *t* = 29.52, *p* < 0.001), Jitter (β = 0.25, se = 0.03, *t* = 7.49, *p* < 0.001), and utterance duration (β = 0.20, se = 0.03, *t* = 6.51, *p* < 0.001), and negatively affected by Shimmer (β = −0.16, se = 0.04, *t* = −4.22, *p* < 0.001), HNR (β = −0.78, se = 0.06, *t* = −12.77, *p* < 0.001), and F1 (β = −0.19, se = 0.03, *t* = −6.20, *p* < 0.001. No effect of F0SD (β < 0.06, se = 0.03, *t* = 1.67, *p* = 0.094) or final word duration (β < −0.02, se = 0.03, *t* = −0.81, *p* = 0.420) were found. As shown in Figure 3, F0M was by far the greatest mediator of Prosody on Response (β = 0.79), followed by shimmer (β = 0.04) and utterance duration (β = 0.02). Meanwhile, the mediations of HNR (β = −0.29), F1 (β = −0.03), and jitter (β = −0.02) were negative. Most of the Prosody effect was not linearly mediated by acoustic measures, as the total indirect effect of Prosody (β = 0.54) accounted for much less of the total effect (β = 2.38). The mediation model is illustrated in Figure 3.

## Discussion

Our results provide experimental evidence supporting the literature on complaints, emotive communication, and vocal affect. As elaborated below, they emphasize the important role of prosody in conveying emotive information in communication and its relationship to other message-level (e.g., lexical) and social-relational (e.g., cultural) dimensions of social interaction.

### Affective Prosody, Effective Complaint

The core of the study measured how listeners evaluate the complaining nature of utterances in different situations combining several factors. The manipulation of the speaker’s prosody was revealed to have the largest effect on listener’s evaluations; everything else controlled for, switching from a “neutral” to a complaining tone of voice led to a marked increase in whether statements were judged to be a complaint. This finding parallels the ability to recognize basic emotions and evaluate speaker arousal from vocal expressions (Scherer, 2003; Grandjean et al., 2006), extending this ability to the general perception of a speaker’s emotivity in discourse. Through the speaker’s intention to foreground speaker affect (Arndt and Janney, 1991; Caffi and Janney, 1994), our results show that complaints can be discriminated from vocal signals without requiring complex attitudinal inferences from situational context (Wichmann, 2000). This exemplifies the use of emotional expressions as a social tool, providing listeners with affective information that allows them to make inferences and to (voluntarily) share the speaker’s emotive state (Van Kleef, 2009; Scarantino, 2017). Here, prosody appears to be the main device in the collaborative treatment of affectivity (Drew, 1998; Selting, 2010), constituting a relatively direct and effective way for listeners to assess a complainer’s subjective state (Acuña-Ferreira, 2002; Edwards, 2005). It can be said that detecting the emotivity of the speaker is a crucial first step in complaining interactions; by allowing listeners to recognize complaints, prosody is likely to play a key role in facilitating the affiliative and empathic response of listeners (Boxer, 1993; Traverso, 2009).

Prosody was not the only way speakers could influence listener’s evaluation of complaints. Utterances that described an explicitly painful situation were perceived as stronger exemplars of complaints than statements which did not. Affective words and sentences are known to affect a listener’s perception of emotions in speech (Pell et al., 2011; Regenbogen et al., 2012; Rigoulot et al., 2020). However, this effect did not interact with prosody and was small enough that control statements spoken in a complaining tone were perceived as more complaining than pain-related sentences in a neutral tone. This confirms an important characteristic of complaints: *how* we complain is more important than *what* we complain about, and one can virtually complain about anything (Alicke et al., 1992; Boxer, 1993). Still, the description of a past situation that would typically be associated with (social) pain can facilitate the perception of an utterance as a complaint; this factor is likely to play a role in how complaining interactions unfold in spontaneous interactions.

Interestingly, the perceptual weight given to the statement seemed to be greater when prosody was less efficient; listeners who were less sensitive to prosodic signals could presumably compensate by relying on the more tangible, explicit nature of verbal information (Zougkou et al., 2017). The relative weighting of prosodic and linguistic information can be partially explained by listener’s empathic abilities; individuals with heightened perspective-taking skills (or *cognitive* empathy), relied more on prosody and less on the verbal statement. In contrast, participants with greater empathic concern (or *affective* empathy) did not show such associations. These results are congruent with the nature of the task, which required *understanding* the speaker’s intention; in this context, the interpretation of the displayed affect would have been driven by inferential rather than affective processes (Van Kleef, 2009). Future research using other designs such as self-ratings or physiological measures (de Vignemont and Singer, 2006; Lang et al., 2011; Kanske et al., 2015) could further distinguish affective from inferential processes in empathy and assess how listeners actually *share* a complainer’s affective state from prosody.

### From Acoustic Signals to Emotive Representations

The manipulation of prosody in our study allowed us to determine to what extent these cues are instrumental for listeners to recognize the speaker’s intent to complain; however, it does not explain *which* acoustic cues drive these judgments and *how* they do it. Prosody researchers who have adopted a Brunswikian approach have stressed that while emotion encoding and decoding have been widely covered by the literature in a separate manner, investigations that *combine* both processes are lacking (Juslin and Laukka, 2003; Scherer, 2003; Grandjean et al., 2006). Acoustic analyses of the present stimuli had revealed a number of parameters that speakers seem to manipulate in order to convey their complaints (Mauchand and Pell, n.d. b, under review). In particular, increased mean F0 and F0 variability, decreased shimmer, increased Harmonics-to-Noise ratio, and lengthened final word were widely used acoustic strategies to communicate complaints. The multiple mediation analysis performed here assessed if and how these parameters were actually used by listeners in their evaluations.

Results of the mediation analysis suggest that mean F0 was by far the most important acoustic parameter in mediating the effect of Prosody; complainers increased their mean pitch, which was perceived as more complaining by listeners. Fundamental frequency is known to be the most directly accessible marker of affect for listeners, and is modulated in both a discrete and continuous manner to express basic emotions (Frick, 1985; Grandjean et al., 2006; Eyben et al., 2016) and attitudes (Jiang and Pell, 2017; Caballero et al., 2018; Mauchand et al., 2018; Truesdale and Pell, 2018). Increased F0 mean also marks non-aggressivity and is central to affiliative behaviors as described by the Frequency code (Ohala, 1984; Gussenhoven, 2004), which could explain its central importance in the production and perception of complaints.

Differences in voice quality also showed notable patterns in mediating the effect of prosody on complaint recognition. Compared to neutral speech, complaints displayed reduced shimmer, increased HNR, and to a lesser extent reduced jitter, indicating that speakers employed a less rough, less creaky and less noisy voice when they were complaining. Evidence of increased voice control (Latoszek et al., 2018) while complaining is also characteristic of simulated but not natural pain (Lautenbacher et al., 2017; Raine et al., 2019). Interestingly, HNR and Jitter negatively mediated the participant’s response, suggesting that listeners may perceive that complaints are not genuine but reconstructed displays of affect (Selting, 2010). This impression may also explain why even complaints with pain-related statements were rarely evaluated using the highest points on the scale. In addition, reduced shimmer was associated with a slight increase in complaint ratings, possibly due to the importance of this acoustic marker in detecting sadness (Juslin and Laukka, 2003). Increased F0SD and Final World Duration, which were associated with complaining prosody, did not significantly affect listener’s judgments in the current study. It should be borne in mind that complaints occur in complex interactions, and the role of prosodic features may not be limited to signaling an emotive intent. Dynamic variations in pitch and rhythm, which mark the emphatic structure of speech (among others), could instead help to coordinate the upcoming interaction and indicate how the collaborative treatment of affect should proceed (Selting, 1994; Szczepek Reed, 2011). Also, the fact that effort-related parameters, such as higher F1 and larger F0 variation (Traunmüller and Eriksson, 2000), had little or even negative effects on the perception of complaints reaffirms that successful complaints are conveyed through affiliative signals (as per the Frequency code), rather than effort-derived meanings (as per the Effort code) (Ohala, 1984; Gussenhoven, 2004).

It is important to note that while a portion of the prosody effect on complaint perception was mediated by specific forms of acoustic change, a large part of the effect remains unexplained in the model. As our selected acoustic parameters cover many of the core acoustic features of utterances (except loudness), it is unlikely that entering more parameters as mediators would significantly increase the proportion of the mediated effect. Instead, it appears that the transformation of acoustic signals into an emotive representation is not a linear process that can be fully decomposed. In the context of our task, it is likely that the apparent contrast between neutral and complaining prosody allowed a discrete categorization of the two utterance types; the relative salience of certain parameters (such as pitch or vocal noise) could then further modulate the perception of utterances within each category.

### Social-Relational Factors in Emotive Communication

While evaluations of complaints relied mainly on prosodic and lexical information, the cultural manipulation of this experiment had a marginal, but still noteworthy, impact on perceptual judgments. Overall, Québécois speakers were rated as producing stronger (i.e., more prototypical) complaints than French speakers, and there was a strong trend for Québécois listeners to recognize complaining prosody better when produced by other Québécois speakers.

In a previous study (Mauchand and Pell, n.d. b, under review), differences between French and Québécois complaints were reported at both the acoustic and perceptual level, motivating our continued interest in how socio-cultural variables influence complaint perception. In that study, we found that Québécois speakers, when complaining, used greater pitch variability and distinct rhythmic patterns than French speakers and were perceived as angrier and more surprised (as opposed to sad for the French speakers, Mauchand and Pell, n.d. b, under review). Of key interest, Québécois speakers used a harsher voice quality than French speakers when producing complaints (reduced HNR). Here, the mediation analysis revealed that HNR reduced the intensity of the perceived complaints; the harsher vocal quality of Québécois speakers might thus have facilitated the detection of complaints by certain listeners. This facilitation was enhanced at the in-group level, as Québécois listeners seemed more attuned to prosodic contrasts produced by other Québécois speakers. This finding suggests the existence of cultural display rules and in-group advantages in emotive speech communication as is the case for the expression of emotions (Elfenbein and Ambady, 2002). However, the absence of a similar in-group advantage for the French group suggests this effect might depend on the interplay of individual, cultural and contextual factors. For example, the exposure of our French participants to the Québécois culture in this study could have reduced potential in-group advantages for that group. However, French participants were very recent immigrants in Québec, and most of them reported having very few Québécois people in their social and professional circles. On the other hand, Québécois participants reported having more French acquaintances, and are frequently exposed to French-accented speech from an early age (Kircher, 2012). Thus, the asymmetry in cultural effects may alternatively be due to a lack of sensitivity of French participants to the more expressive Québécois complaining style.

While the decoding of emotive cues in the voice may be enhanced for certain in-group interactions, this facility does not seem to originate from cultural bias or prejudice. No relationship was found between the effect of accent and either implicit or explicit biases toward either group, even though such biases exist between French and Québécois communities (Auger and Valdman, 1999; Kircher, 2012; Mauchand and Pell, n.d. a, under review). While stereotypes and prejudice do affect neural activity (Quadflieg and Macrae, 2011; Jiang et al., 2018) and affective empathy (Xu et al., 2009; Contreras-Huerta et al., 2014), they often don’t impede speech comprehension and affect recognition (Gill, 1994; Lev-Ari and Keysar, 2010; Thierry et al., 2015). Thus, accent effects may instead arise from processing issues and/or differential use of prosodic signals. Even then, the potential impact of accent cues were minimal when compared to the efficacy of both speaker groups to convey a complaining intention through prosody. These results thus reveal a strong consistency of speakers in intentionally using emotions as social signals and of listeners to infer their intentions in the case of complaints. This inference process can be subtly modulated by social-relational factors, such as the culturally normative usage of certain prosodic cues (Elfenbein and Ambady, 2002; Laukka et al., 2016; Scherer, 2003; Van Kleef, 2009). Other factors not taken into account here may also play an important role in natural complaint perception: here, the absence of context, visual cues, or a true indication of the social proximity between speaker and listener might explain why evaluations of complaints rarely reached the end of the scale. Sex/gender is also often mentioned as an important factor in complaining (Acuña-Ferreira, 2002; Selting, 2010); anecdotally, speaker sex was tentatively added as a parameter in our model, but did not show any significant effect (although this could be due to the small number of male/female speakers in our experiment). Future studies should investigate how a wider range of these social factors influence inferential and affective processes underlying emotive speech communication.

## Conclusion

As the first perceptual investigation of complaining speech, the present study reaffirms the central role of prosody as a social device to foreground the emotive state of the speaker. The effective production and appraisal of emotive features in the voice denote a tacit understanding between speaker and listener on how complaints are performed, which depends on the capacity of listeners to detect these signals and collaborate with the social goals of the speaker (i.e., to commiserate and co-complain). Listeners also use linguistic evidence describing the nature and/or antecedents of a complaint when evaluating these speech acts, although these cues may be less diagnostic than prosodic contrasts for determining when a speaker intends to complain (and seek social affiliation and support). As such, complaints can be qualified as acts of manipulation without deception, similar to other emotive acts like persuasion, motivation or charismatic speech: intentional displays of emotion that regulate the dispositional affect of listeners and promote social affiliation. This metapragmatic understanding of human affect, central to speaker/listener relationships, needs to be systematically considered in future investigations of speech, attitudes, and emotions (Pell and Kotz, 2020). Including social-relational factors, such as cultural relationships, is crucial to advance perspectives in this literature; future work should investigate how more distant cultures communicate complaints and other types of emotive meanings. Experimental approaches that include empathic assessments, neurophysiological measures, or which study group interactions would also produce valuable evidence to build on theoretical frameworks describing emotive communication, affect, and prosody.

## Data Availability Statement

The datasets presented in this study can be found in online repositories. Data and materials can be found on the OpenScience Framework (Foster and Deardorff, 2017; https://osf.io/9az68/?view_only=9a5529fc1a0645aa9b17f6589a79e848).

## Ethics Statement

The studies involving human participants were reviewed and approved by the Faculty of Medicine and Health Sciences Institutional Review Board (McGill IRB). The patients/participants provided their written informed consent to participate in this study.

## Author Contributions

MM designed the experiment, collected, analyzed and interpreted data, and drafted and developed the manuscript. MP supervised the experiment and provided critical revisions of the manuscript. Both authors contributed to the article and approved the submitted version.

## Conflict of Interest

The authors declare that the research was conducted in the absence of any commercial or financial relationships that could be construed as a potential conflict of interest.

## Appendix

**TABLE A1:** Sentences constructed for the experiment with English translations. Only the bolded final words differed between the Pain and Control version of one sentence root.

Experimental Stimuli		*English translation*	
Pain	Control	*Pain*	*Control*
Ils ont tout fait sans **moi**	Ils ont tout fait sans **boire**	*They did everything without me*	*They did everything without drinking*
Ils sont partis sans **moi**	Ils sont partis sans **barque**	*They left without me*	*They left without a boat*
Ils m’ont demandé de **partir**	Ils m’ont demandé de **rester**	*They asked me to leave*	*They asked me to stay*
Ils refusé de venir chez **moi**	Ils ont refusé venir chez **Marc**	*They decided not to come to my place*	*They decided not to come to Marc’s*
Ils ont refusé **m’inviter**	Ils ont refusé de **mélanger**	*They refused to invite me*	*They refused to stir*
Ils ont décidé de pas **m’inviter**	Ils ont décidé de pas **mélanger**	*They decided not to invite me*	*They decided not to stir*
Ils ont décidé de pas jouer avec **moi**	Ils ont décidé de pas jouer avec **Marc**	*They decided not to play with me*	*They decided not to play with Marc*
Ils ont décidé d’y aller sans **moi**	Ils ont décidé d’y aller sans **masques**	*The decided to go without me*	*They decided to go without masks*
Il m’a choisi en **dernier**	Il m’a choisi en **deuxiéme**	*He chose me last*	*He chose me in second*
Elle veut que personne **m’aime**	Elle veut que personne **marche**	*She wants no one to like me*	*She wants no one to walk*
Il me parle **jamais**	Il me parle jeudi	*He never talks to me*	*He talks to me thursday*
Elle a profité de **moi**	Elle a profité de **l’offre**	*She took advantage of me*	*She took advantage of the sale*
Il m’a fait **pleurer**	Il m’a fait **parler**	*He made me cry*	*He made me talk*
Elle m’a répondu **méchamment**	Elle m’a répondu **normalement**	*He answered me harshly*	*He answered me normally*
Elle a dit qu’elle m’aimait **pas**	Elle a dit qu’il m’aimait **bien**	*She said he didn’t like me*	*She said he appreciated me*
Il veut vraiment pas de **moi**	Il veut vraiment pas de **masque**	*He really doesn’t want me*	*He really doesn’t want a mask*
Elle continue de **m’ignorer**	Elle continue de **mesurer**	*She keeps ignoring me*	*She keeps measuring*
Il a dit que j’étais **stupide**	Il a dit que j’étais **sorti**	*He said I was stupid*	*He said I went out*
Elle a dit que j’étais **sale**	Elle a dit que j’étais **jeune**	*She said I was dirty*	*She told me I was young*
Il a dit que j’étais **gros(se)**	Il a dit que j’étais **grand**	*He said I was fat*	*He said I was tall*
Elle pense que je suis **mauvaise**	Elle pense que je suis **bronzé**	*She thinks I am bad*	*She thinks I am tan*
Ils pensent que je suis **méchant(e)**	Ils pensent que je suis **belge**	*They think I am mean*	*They think I am belgian*
Elle pense que je suis **peureux (se)**	Elle pense que je suis **parti(e)**	*Shee thinks I am scared*	*She thinks I am gone*
Il pense que je suis **faible**	Il pense que je suis **fier**	*He thinks I am weak*	*He thinks I am proud*
Ils font des blagues sur **moi**	Ils font des blagues sur **Mars**	*They made jokes about me*	*They make jokes about Mars*
Ils font des blagues sur **mon poids**	Ils font des blagues sur **Montréal**	*They make jokes about my weight*	*They make jokes about Montreal*
Elle déteste **mon idée**	Elle déteste **mélanger**	*She hates my idea*	*She hates stirring*
Il m’a fait passer pour un **fou**	Il m’a fait passer pour un **frére**	*He made me look like madman*	*He made me look like a brother*
Elle m’a donné une **claque**	Elle m’a donné une **glace**	*Shee gave me a slap*	*She gave me an ice cream*
Il m’a donné un coup de **pied**	Il m’a donné un coup de **main**	*He gave me a kick*	*He gave me help*
Il m’empêche de **m’amuser**	Il m’empêche de **glisser**	*He prevents me from having fun*	*He rpevents me from slipping*
Elle m’empêche de **dormir**	Elle m’empêche de **tomber**	*She prevent me from sleeping*	*She prevents me from falling*
Il continue de me **mentir**	Il continue de me **montrer**	*He keeps lying to me*	*He keeps showing me*
Elle continue de **m’insulter**	Elle continue de **mélanger**	*She keeps insulting me*	*She keeps stirring*
Il a marché sur **mon chien**	Il a marché sur **mon chemin**	*He stepped on my dog*	*He stepped on my path*
Elle a marché sur **ma main**	Elle a marché sur **ma route**	*She stepped on my hand*	*She stepped on my road*
Il a frappé **ma jambe**	Il a frappé **ma balle**	*He kicked my leg*	*He kicked my ball*
Elle a pris **ma place**	Elle a pris **ma main**	*She took my spot*	*She took my hand*
Il m’a fait **tomber**	Il m’a fait **comprendre**	*He made me fall*	*He amde me understand*
Elle veut me faire **rater**	Elle veut me faire **rester**	*She wants to make me fail*	*She wants to make me stay*
Il essaye de **m’énerver**	Il essaye de **mesurer**	*He’s trying to annoy me*	*He’s trying to measure*
